# SAFE AND EFFECTIVE PAIN CONTROL FOR GERIATRIC PATIENTS: A MULTIDISCIPLINARY APPROACH DURING THE OPIOID EPIDEMIC

**DOI:** 10.1093/geroni/igz038.1490

**Published:** 2019-11-08

**Authors:** Tatyana Gurvich

**Affiliations:** University of Southern California, Los Angeles, California, United States

## Abstract

Opioid use is at a crisis level. According to the CDC, an estimated 20 % of patients presenting to physician offices with non-cancer pain receive an opioid prescription (1). According to the Administration on Aging and Substance Abuse and Mental Health Services Administration, the population of older adults who misuse opioids is expected to double by 2020. Today’s mandate to reduce opioid use and to manage patients safely with adjuvant medications comes with many challenges in geriatrics. Many patients have comorbidities which limit the use of adjuvant pain medications. A careful balance must be achieved, in order to provide good pain management and improve quality of life in this patient population. This symposium will explore multidisciplinary approaches to managing pain in geriatrics to reduce opioid use and manage safe opioid use where necessary. Pharmacological strategies for adjusting dosing and managing compliance will be discussed. Cooperative education to improve prescribing practices along with patient education to improve safe use, are important elements. Adjunct use of physical therapy and integrative medicine are also discussed as viable and effective adjuncts or alternatives to traditional pain management. You will learn how to use medications safely, utilize physical therapy to its maximum potential and learn more about innovative integrative medicine techniques, all of which decrease pain and improve function and most importantly quality of life. (1) Daubresse M, Chang HY, Yu Y, et al. Ambulatory diagnosis and treatment of nonmalignant pain in the United States, 200-2010. Med Care 2013; 51:870-8

